# Cognitive Behavioral Therapy Reduces Symptom Severity and Normalizes Neurophysiological and Attentional Reactivity in Anorexia Nervosa: A Randomized Controlled Trial

**DOI:** 10.3390/brainsci16030309

**Published:** 2026-03-13

**Authors:** Eda Yılmazer, Metin Çınaroğlu, Selami Varol Ülker, Gökben Hızlı Sayar

**Affiliations:** 1 Psychology Department, Faculty of Social Science, Beykoz University, Istanbul 34805, Türkiye; edayilmazer@beykoz.edu.tr; 2 Psychology Department, Faculty of Administrative and Social Science, İstanbul Nişantaşı University, Maslak Mah. Taşyoncası Sok. No: 1V ve No:1Y Sarıyer, Istanbul 34398, Türkiye; 3 Faculty of Humanities and Social Sciences, Üsküdar University, Istanbul 34662, Türkiye; selamivarol.ulker@uskudar.edu.tr; 4 Medical School, Üsküdar University, Istanbul 34662, Türkiye; gokben.hizlisayar@uskudar.edu.tr

**Keywords:** anorexia nervosa, cognitive behavioral therapy, EEG, eye-tracking, galvanic skin response, attentional bias, emotion regulation, randomized controlled trial, eating disorders

## Abstract

Background: Anorexia nervosa (AN) is a severe psychiatric disorder marked by restrictive eating, distorted body image, and high relapse rates. While cognitive-behavioral therapy (CBT) is a widely used treatment, its mechanisms of action in AN remain incompletely understood, particularly beyond self-reported symptom change. This study investigated the effects of a 12-week CBT intervention on both clinical and multimodal laboratory-based outcomes in women with restrictive-type AN. Methods: In a two-arm, pre–post randomized controlled trial (ClinicalTrials.gov: NCT07037017), 59 women with restrictive-type AN were randomized to a CBT intervention (*n* = 30) or no-treatment control (*n* = 29). A total of 50 participants (CBT: 26; control: 24) completed baseline and post-intervention assessments and were included in analyses. Outcomes included psychometric measures (eating disorder symptoms, depression, anxiety, body image-related obsessive–compulsive symptoms, and cognitive emotion regulation) and laboratory-based indices: electroencephalography (EEG), galvanic skin response (GSR), and eye-tracking during exposure to food- and body-related stimuli. Group × Time effects were analyzed using repeated-measures mixed-effects models, and statistical analyses were conducted using SPSS (Version 31; IBM Corp., Armonk, NY, USA). Results: Significant Group × Time interactions indicated greater improvements in the CBT group across all psychometric outcomes, including reduced eating disorder symptom severity (*p* < 0.001, η_p_^2^ = 0.28) and increased adaptive emotion regulation. CBT participants also showed significant reductions in EEG P300 and late positive potential (LPP) amplitudes to body-related stimuli, increased frontal alpha asymmetry, decreased visual fixation on salient body and food cues, and attenuated GSR reactivity (all *p* < 0.05). Exploratory correlations revealed that symptom improvements were associated with reductions in neurophysiological and attentional reactivity. Conclusions: To our knowledge, this is the first RCT in AN to demonstrate that CBT not only improves self-reported outcomes but also modulates neurophysiological and attentional processes implicated in the maintenance of the disorder. Multimodal laboratory assessments provided mechanistic insight into treatment effects and may inform personalized intervention strategies. CBT appears to facilitate recovery through both cognitive–emotional and physiological recalibration.

## 1. Introduction

Anorexia nervosa (AN) is a serious psychiatric illness characterized by extreme dietary restriction, low body weight, and distorted body image [1]. It predominantly affects young women, with epidemiological studies indicating a prevalence ranging from approximately 0.9% up to 4% of females (and around 0.3% of males) in Western countries [2]. AN has one of the highest mortality rates among mental disorders [3]. Medical complications from prolonged malnutrition—including cardiovascular, gastrointestinal, and endocrine sequelae—are common and can cause lasting organ damage even after weight recovery [4]. Approximately 6% of patients die within four years of initial diagnosis, underscoring the grave prognosis if the illness remains untreated or poorly managed [5]. Beyond mortality, AN often follows a chronic, relapsing course; it is estimated that over half of individuals who initially recover will relapse in the years following treatment [6]. This combination of high morbidity, mortality, and relapse risk highlights the urgent need for effective, sustained interventions for AN.

Neurobiological research has increasingly highlighted a complex and, at times, paradoxical pattern of brain functioning in anorexia nervosa (AN), characterized by heightened limbic responsivity alongside pronounced behavioral inhibition. Functional neuroimaging studies using food-cue exposure paradigms consistently report increased activation in limbic and paralimbic regions, including the amygdala, anterior insula, and anterior cingulate cortex, particularly when participants are presented with high-calorie food stimuli or disorder-relevant body images [7,8]. Effect sizes in these studies are often in the moderate range (e.g., Cohen’s d ≈ 0.5–0.8 for amygdala and insula hyperactivation compared to healthy controls), suggesting robust emotional salience attribution to disorder-specific cues. Paradoxically, despite this elevated neural reactivity, individuals with AN demonstrate marked behavioral inhibition and restrictive control over food intake. This pattern has been interpreted within a dual-process framework, in which heightened bottom-up limbic threat signaling is counterbalanced by exaggerated top-down cognitive control mediated by prefrontal networks. Tasks involving cognitive reappraisal, inhibitory control (e.g., Go/No-Go or Stroop paradigms), and delayed reward processing often reveal increased dorsolateral prefrontal cortex engagement and altered fronto-striatal connectivity, supporting the hypothesis of overregulated affective responding rather than impulsivity.

Importantly, many neuroimaging samples include individuals with comorbid depression, anxiety disorders, or obsessive–compulsive symptoms—conditions independently associated with altered limbic and prefrontal activation patterns. These comorbidities may amplify amygdala hyperreactivity or modulate prefrontal control mechanisms, complicating interpretation of disorder-specific effects. Moreover, task characteristics significantly influence observed activation patterns. For example, passive viewing of food images typically elicits greater insula and amygdala activation, whereas tasks requiring explicit emotion regulation engage prefrontal regions more strongly. Eye-tracking and psychophysiological studies similarly report moderate attentional bias effects toward weight- and shape-related stimuli (η^2^ ≈ 0.10–0.20), alongside elevated autonomic reactivity measured via skin conductance response. Together, this literature suggests that AN is characterized not merely by heightened emotional sensitivity to disorder-relevant cues, but by a maladaptive interaction between affective salience and rigid cognitive control. These findings provide a neurobiological rationale for interventions, such as cognitive-behavioral therapy, that aim to recalibrate both emotional reactivity and cognitive regulation processes.

Delayed identification and treatment initiation remain significant clinical challenges in anorexia nervosa. Several studies indicate that a substantial period may elapse between initial symptom onset and formal diagnosis, particularly in restrictive-type presentations where weight loss may be gradual and ego-syntonic beliefs about control and thinness are reinforced [5,6]. During this interval, symptoms often become entrenched, contributing to chronicity and poorer treatment response. Furthermore, anorexia nervosa is frequently accompanied by comorbid psychiatric conditions, including major depressive disorder, anxiety disorders, and obsessive–compulsive spectrum symptoms [3,7]. These comorbidities may mask core eating disorder pathology or lead clinicians to initially prioritize mood or anxiety symptoms, thereby delaying accurate diagnosis and disorder-specific intervention. The presence of comorbidity is also associated with greater illness severity and functional impairment, underscoring the importance of comprehensive assessment and early, mechanism-informed treatment approaches.

Evidence-based treatments for eating disorders include several psychotherapeutic approaches. For adolescent AN, family-based treatment is a first-line intervention, whereas for adults, individualized therapies are commonly used [9]. Among these, cognitive behavioral therapy (CBT) is one of the most widely utilized treatments across eating disorders [10,11]. Enhanced cognitive-behavioral therapy (CBT-E), a transdiagnostic form of CBT tailored for eating disorders, targets maintaining mechanisms such as overvaluation of weight/shape, rigid dietary restraint, and emotional avoidance [12]. National clinical guidelines frequently recommend CBT as a cornerstone of outpatient treatment for eating disorders [13]. However, the efficacy of CBT in anorexia nervosa has been less definitively established than in bulimia nervosa or binge-eating disorder. A recent meta-analysis noted a paucity of randomized trials focusing on AN, finding that there were not enough studies to draw robust conclusions on CBT’s effectiveness in this population [14]. Unlike in bulimia, where CBT consistently outperforms waitlist controls, trials in AN often use active comparison therapies and have yielded mixed results. CBT has not demonstrated clear superiority over other psychotherapies for AN, and treatment response tends to be variable [15]. In practice, many adult AN patients show only partial remission of symptoms, and dropout rates from therapy can be high [16]. Furthermore, long-term outcomes remain disappointing, with high relapse rates despite initial symptom improvement [17]. These challenges suggest that while CBT is a central and promising treatment, there is room to enhance its impact and better understand for whom and how it works in anorexia nervosa.

One critical limitation in the current literature is the relative lack of studies examining objective neurophysiological and cognitive-behavioral processes that underlie symptom change in AN treatments. Anorexia nervosa is associated with distinct neurocognitive disturbances, such as heightened emotional reactivity to food-related stimuli and cognitive inflexibility around body image [7]. For example, individuals with AN exhibit amplified activity in limbic regions like the amygdala when exposed to food or body cues and demonstrate attentional biases toward disorder-relevant stimuli [18]. They unconsciously prioritize food and body-related cues, which can manifest as distraction or anxiety during tasks [8]. These attentional biases and alterations in fronto-limbic circuitry—including heightened amygdala and insula reactivity to disorder-relevant cues and altered prefrontal regulatory engagement—are thought to contribute to the onset and maintenance of the illness. However, the precise cognitive-emotional mechanisms in AN remain incompletely understood, and importantly, most treatment trials do not incorporate measures to directly observe changes in these underlying processes. The focus of prior RCTs has largely been on clinical outcomes (weight gain, eating disorder psychopathology scores, etc.), without assessing whether therapy produces measurable changes in the brain or behavior beyond self-report [19,20].

In related psychiatric conditions, researchers have highlighted the value of a multimodal approach to understand therapy mechanisms. By integrating neurophysiological, psychophysiological, and behavioral assessments, one can begin to link subjective improvements to objective changes in underlying systems. In anorexia, to the best of our knowledge, no published randomized trial to date has combined traditional psychometric outcomes with measures like electroencephalography (EEG), galvanic skin response (GSR), and eye-tracking to capture neurocognitive and physiological changes during therapy. This represents a significant gap in knowledge. Addressing this gap is important because the success of CBT in AN likely hinges not only on weight restoration and self-reported symptom reduction, but also on modifying maladaptive information-processing patterns (e.g., attentional fixation on body flaws, anxiety responses to food) and improving emotion regulation capacity. Objective biomarkers—for instance, changes in event-related brain potentials, autonomic arousal to stimuli, or visual gaze patterns—could reveal whether therapy is truly altering the disordered cognitive-affective processes characteristic of AN. Multimodal lab-based assessments can thus provide a window into the mechanistic effects of treatment that is not accessible through questionnaires alone. Indeed, recent work in body image disorders has called for integrating such measures, noting that understanding interactions between psychological symptoms and physiological responses can guide more effective interventions [21,22,23].

The present study aims to innovate in this direction by evaluating a 12-week CBT intervention for restrictive-type anorexia nervosa using a comprehensive battery of clinical and laboratory measures. We conducted a two-arm randomized controlled trial with an active CBT group versus a no-treatment control, in a pre–post design (ClinicalTrials.gov NCT07037017). In addition to standard clinical assessments (eating disorder symptom severity, comorbid anxiety and depression, body image-related obsessive-compulsive symptoms, and emotion regulation strategies), participants underwent laboratory evaluations of brain, autonomic, and attentional responses. Specifically, EEG was recorded during exposure to food- and body-related stimuli to index neural processing (including components such as P300 and Late Positive Potential [LPP] reflecting attention and emotional salience, and frontal alpha asymmetry reflecting emotional regulation). Simultaneously, eye-tracking measured visual attention biases (e.g., fixation duration and gaze patterns toward high-calorie food images and body shape regions), and GSR captured physiological arousal (skin conductance changes) in response to these stimuli. By integrating these modalities, our study provides a novel multimodal examination of CBT’s effects in AN, enabling us to test whether subjective improvements correspond with objective changes in neurophysiology and behavior.

We hypothesized that the CBT intervention would lead to greater improvements than the control condition across both psychometric and lab-based outcomes. Specifically, we anticipated that women receiving 12 weeks of CBT would show significant reductions in eating disorder psychopathology (improved Eating Disorder Examination-Questionnaire [EDE-Q] scores), as well as decreases in comorbid anxiety and depressive symptoms, and improvements in adaptive emotion regulation, relative to controls. We further expected objective evidence of therapeutic change in the CBT group: namely, reduced EEG markers of hyper-responsivity to disorder-related cues (e.g., lower P300/LPP amplitudes to food and body stimuli), lower autonomic arousal to these cues (smaller skin conductance responses), and attenuation of attentional biases, such as less visual fixation on feared body regions or high-calorie foods. By contrast, minimal changes were expected in the control group. By clearly demonstrating both the clinical efficacy of CBT and its concomitant neurophysiological and attentional effects, this study seeks to advance our understanding of the mechanisms through which CBT may benefit individuals with anorexia nervosa. Ultimately, this multimodal RCT approach is positioned to inform more targeted therapies and improved outcome monitoring in AN, bridging the gap between subjective symptom change and objective biological markers of recovery.

## 2. Materials and Methods

### 2.1. Study Design

This study was conducted as a registered clinical trial (ClinicalTrials.gov Identifier: NCT07037017) for evaluating the effect of Cognitive Behavioral Therapy on anorexia nervosa symptoms among women using a two-arm, pre–post controlled design. Following baseline (pre-intervention) assessments, participants were randomly allocated to either a cognitive–behavioral therapy (CBT) intervention group (*n* = 30) or a control group (*n* = 30) using a computer-based randomization program administered by a research assistant under the supervision of the lead author. All participants completed identical psychological and laboratory-based assessments at baseline and again after the 12-week period. After completion of baseline assessments, participants in the CBT group received a 12-week CBT intervention, whereas participants in the control group did not receive any psychological intervention during the study period. Psychological outcomes were evaluated using validated self-report measures assessing eating disorder symptom severity, anxiety, depression, body image–related obsessive–compulsive features, and cognitive emotion regulation. In addition to psychometric assessments, laboratory-based measures were employed to capture objective indices of neurophysiological, physiological, and attentional processing, including electroencephalography (EEG), galvanic skin response (GSR), and eye-tracking during standardized visual tasks. This combined pre–post clinical and experimental framework enabled comparison of changes over time between the CBT and control groups and was designed to evaluate both subjective symptom change and underlying psychophysiological mechanisms associated with the intervention.

### 2.2. Participants and Recruitment

Participants (all female) were recruited from the outpatient psychiatric clinic of one of the study authors, a psychiatrist (G.H.S.), as well as from outpatient clinics of collaborating psychiatrists working in governmental hospitals and private clinical settings in Istanbul, Türkiye. In total, 74 individuals were assessed for eligibility. Fifteen individuals were excluded during screening: six did not meet DSM-5 criteria for restrictive-type anorexia nervosa, three met criteria for another eating disorder (e.g., binge–purge subtype or bulimia nervosa), two had comorbid bipolar disorder, two had substance use disorders, and two were excluded due to neurological or medical conditions incompatible with laboratory-based assessments. The remaining 59 participants met all inclusion criteria and were enrolled and randomized (CBT: *n* = 30; control: *n* = 29). Based on the a priori sample size calculation, the target enrollment was 60 participants; thus, the final enrolled sample (*n* = 59) was considered sufficient to maintain the planned statistical power.

Eligible participants were adults aged 18–35 years who met DSM-5 diagnostic criteria for restrictive-type anorexia nervosa within the preceding 6–12 months. Psychiatric diagnoses and comorbidities were assessed using a structured DSM-5–based clinical interview conducted by the recruiting psychiatrist. Diagnostic determinations were supported by review of clinical records where available and independently verified by two assistant professors of clinical psychology to ensure diagnostic reliability and accurate application of inclusion and exclusion criteria.

Participants were required to be medically stable, capable of completing psychological assessments and laboratory-based procedures, and willing to participate in the full intervention period and all pre- and post-intervention assessments, providing written informed consent prior to enrollment. Individuals were excluded if they had a current or lifetime diagnosis of other eating disorders, severe comorbid psychiatric conditions (e.g., psychotic disorders, bipolar disorder, or substance use disorders), a history of neurological disease or traumatic brain injury, or visual, cognitive, or neurological impairments that could interfere with EEG, eye-tracking, or GSR recordings. Additional exclusion criteria included current pregnancy and the use of psychotropic medications that could affect neurophysiological or psychophysiological measurements.

During the study, nine participants discontinued participation. In the CBT group, four participants withdrew due to health-related or personal reasons unrelated to the intervention. In the control group, five participants withdrew, primarily due to non-attendance at post-intervention laboratory sessions and/or failure to complete post-intervention psychometric assessments. Despite these withdrawals, the overall sample size (*n* = 50) planned for analysis was retained, and all participants with complete baseline and post-intervention data were included in the final analyses.

### 2.3. Intervention/Experimental Procedure

The cognitive–behavioral therapy (CBT) intervention was delivered by four study authors, including three assistant professors of clinical psychology and one professor of psychiatry, all of whom had a minimum of 10 years of clinical experience in CBT. The intervention followed a structured and manualized CBT-E–based protocol and was administered over a 12-week period.

Treatment fidelity and adherence were monitored through structured supervision procedures led by an independent professor of psychiatry (M.B.), who was not involved in treatment delivery. All therapists followed the same manualized protocol and completed standardized session checklists documenting the implementation of core CBT components (e.g., psychoeducation, self-monitoring, cognitive restructuring, exposure exercises, and relapse prevention planning). Weekly supervision meetings were conducted to review case formulations, session structure, and protocol adherence across therapists. In addition, a randomly selected subset of session materials (approximately 20%) was reviewed to evaluate consistency with the manualized intervention. No major deviations from the protocol were identified.

In addition to the clinical intervention, participants completed standardized laboratory-based experimental assessments at baseline and post-intervention. These assessments were conducted in a controlled laboratory environment and included electroencephalography (EEG), galvanic skin response (GSR), and eye-tracking recordings while participants were exposed to standardized visual stimuli related to body image, food, and self-appearance. The laboratory procedures were identical across assessment time points and were administered by trained research staff using standardized protocols to ensure consistency and reliability of data collection.

### 2.4. Intervention Protocol

The intervention consisted of a manualized, disorder-specific cognitive–behavioral therapy program based on enhanced cognitive behavioral therapy (CBT-E) as described by Fairburn (2008) [24]. The treatment followed the core structure and therapeutic principles outlined in the CBT-E manual for eating disorders and was not developed de novo for the present study. The protocol was adapted to a 12-session format consistent with focused CBT-E delivery, while maintaining the essential components and sequencing specified in the published treatment model. The intervention was delivered over 12 consecutive weeks, with one individual session per week lasting approximately 60 min, and was conducted online via Microsoft Teams (Version 2412; Microsoft Corporation, Redmond, WA, USA).

The CBT-E framework was selected due to its strong empirical support for eating disorders and its transdiagnostic focus on maintaining mechanisms, including overvaluation of weight and shape, rigid dietary restraint, perfectionistic control strategies, and maladaptive coping processes.

The intervention followed a phased structure consistent with the CBT-E model. The initial phase emphasized psychoeducation, collaborative case formulation, establishment of regular eating patterns, and structured self-monitoring of eating behaviors and associated cognitions. The middle phase targeted core maintaining mechanisms through cognitive restructuring of dysfunctional beliefs related to weight, shape, and control; behavioral experiments addressing avoidance and safety behaviors; graduated exposure to feared foods; and development of adaptive emotion regulation strategies aimed at reducing reliance on restrictive behaviors. The final phase focused on relapse prevention, consolidation of therapeutic gains, and development of individualized maintenance plans to support sustained improvement following completion of the intervention. No additional psychological interventions were introduced during the treatment period.

### 2.5. Experimental Task

All laboratory-based experimental procedures were conducted at the Üsküdar University Neuro-Psychology Laboratory in İstanbul, led by one of the study authors (S.V.Ü.). Experimental assessments were administered at both baseline (pre-intervention) and post-intervention, following identical protocols across time points.

The experimental task was implemented using iMotions software (version 10.1.1; iMotions A/S, Copenhagen, Denmark), which enabled synchronized acquisition of multimodal psychophysiological and behavioral data. Visual stimuli related to body image (self and other female bodies) and food (low- and high-calorie items) were presented on a computer screen in randomized order while participants were seated in a quiet, dimly lit laboratory environment.

Electroencephalography (EEG) data were recorded continuously using the B-Alert X-24 EEG system (Advanced Brain Monitoring Inc., Carlsbad, CA, USA), allowing assessment of neural indices of attentional and emotional processing. Task-locked EEG recordings were used to derive event-related potentials, including the P300 and late positive potential (LPP), as well as frontal alpha asymmetry measures associated with affective regulation.

Eye-tracking data were collected using the Gazepoint GP3 eye tracker (Gazepoint Research Inc., Vancouver, BC, Canada) to quantify visual attention patterns during stimulus presentation. Fixation duration and gaze distribution were extracted for predefined areas of interest, particularly weight- and shape-related body regions and food-related stimuli, to assess attentional biases relevant to eating disorder psychopathology.

Autonomic arousal was assessed via galvanic skin response (GSR) using the Shimmer3 GSR+ system (Shimmer Research Ltd., Dublin, Ireland). Skin conductance responses were recorded continuously during the task to capture physiological reactivity to body- and food-related visual stimuli.

In addition, facial affective responses were analyzed using Affdex facial coding integrated within the iMotions platform. Facial muscle activity was automatically classified into basic emotional expressions and valence–arousal dimensions during stimulus exposure. All data streams were temporally synchronized within the iMotions environment, and acquisition parameters were kept constant across participants and assessment sessions to ensure data consistency and comparability.

### 2.6. Measures

#### 2.6.1. Sociodemographic and Anthropometric Data

Sociodemographic information was collected using a structured self-report form and included age, sex, educational level, marital status, and employment status. Anthropometric data comprised self-reported body weight and height, from which body mass index (BMI; kg/m^2^) was calculated. All sociodemographic and anthropometric variables were assessed at baseline and used to characterize the study sample and, where appropriate, as covariates in subsequent analyses.

#### 2.6.2. Eating Disorder Examination Questionnaire (EDE-Q)

Eating disorder symptom severity was assessed using the Eating Disorder Examination Questionnaire (EDE-Q), a self-report instrument developed by Fairburn and Beglin (1994) [25] as the questionnaire version of the Eating Disorder Examination interview. The EDE-Q consists of 28 items assessing eating disorder psychopathology over the past 28 days across four subscales: Restraint, Eating Concern, Shape Concern, and Weight Concern, yielding a global score. The Turkish version of the EDE-Q was validated by Yucel et al. (2011) [26] in a large adolescent sample, demonstrating good psychometric properties. Internal consistency for the Turkish version was high, with a Cronbach’s α of 0.93 for the total score, and subscale α values ranging from 0.70 to 0.86, supporting its reliability for use in Turkish populations.

#### 2.6.3. Beck Depression Inventory-II (BDI-II)

Depressive symptom severity was measured using the Beck Depression Inventory-II (BDI-II), developed by Beck et al. (1996) [27] as a 21-item self-report scale assessing cognitive, affective, and somatic symptoms of depression over the past two weeks. The BDI-II has been widely used in both clinical and research settings. The Turkish adaptation and validation of the Beck Depression Inventory was conducted by Kapci et al. (2008) [28], demonstrating satisfactory psychometric properties in psychiatric and non-psychiatric samples. The Turkish version showed good internal consistency, with reported Cronbach’s α values ranging from 0.74 to 0.80, indicating acceptable reliability for assessing depressive symptoms.

#### 2.6.4. Beck Anxiety Inventory (BAI)

Anxiety symptoms were assessed using the Beck Anxiety Inventory (BAI), originally developed by Beck et al. (1988) [29] to measure the severity of somatic and cognitive symptoms of anxiety. The BAI consists of 21 items, each rated on a 4-point Likert scale. The Turkish version of the BAI was validated by Ulusoy et al. (1998) [30] in psychiatric outpatient samples. The Turkish adaptation demonstrated excellent internal consistency, with a reported Cronbach’s α of 0.93, supporting its reliability and validity for use in Turkish clinical populations.

#### 2.6.5. Yale-Brown Obsessive Compulsive Scale—Body Dysmorphic Disorder Version (YBOCS-BDD)

Body image-related obsessive–compulsive symptom severity was evaluated using the Yale-Brown Obsessive Compulsive Scale modified for Body Dysmorphic Disorder (YBOCS-BDD), originally developed by Phillips et al. (1966) [31]. This 12-item, clinician-rated scale assesses the severity of preoccupations and compulsive behaviors related to perceived appearance defects over the previous week. The Turkish version of the YBOCS-BDD was validated by Yücesoy et al. (2022) [32], demonstrating good psychometric properties. The Turkish adaptation showed strong internal consistency, with a Cronbach’s α of 0.81, supporting its reliability for assessing body dysmorphic symptoms in Turkish samples.

#### 2.6.6. Cognitive Emotion Regulation Questionnaire (CERQ)

Cognitive emotion regulation strategies were assessed using the Cognitive Emotion Regulation Questionnaire (CERQ), developed by Garnefski et al. (2001) [33] to measure cognitive coping strategies following negative life events. The CERQ consists of 36 items across nine subscales, including rumination, catastrophizing, positive reappraisal, and refocus on planning. The Turkish version of the CERQ was validated by Tuna and Bozo (2012) [34] in a university student sample. The Turkish adaptation demonstrated good internal consistency, with Cronbach’s α values for subscales ranging between 0.72 and 0.83, indicating satisfactory reliability for assessing cognitive emotion regulation strategies in Turkish populations.

### 2.7. Physiological/Behavioral/Laboratory Assessments

#### 2.7.1. Electroencephalography (EEG)

Neural activity was recorded using a B-Alert X-24 EEG system, which allows multichannel, mobile electroencephalographic recording during experimental tasks. EEG data were collected continuously while participants were exposed to standardized visual stimuli related to body image and food. Recordings were conducted in accordance with international EEG recording guidelines, and electrode placement followed a standardized scalp montage. Event-related potentials (ERPs) associated with attentional and emotional processing, including the P300 and late positive potential (LPP), as well as frontal alpha asymmetry, were derived from task-locked EEG data to index attentional salience, emotional reactivity, and affect regulation.

#### 2.7.2. Eye-Tracking

Visual attention patterns were assessed using the Gazepoint GP3 eye-tracking system. Eye-tracking data were recorded concurrently with stimulus presentation to quantify gaze behavior during exposure to body- and food-related images. Primary eye-tracking outcomes included fixation duration, fixation count, and gaze distribution across predefined areas of interest (AOIs), particularly weight- and shape-related body regions and food stimuli. These measures were used to assess attentional biases relevant to eating disorder psychopathology.

#### 2.7.3. Galvanic Skin Response (GSR)

Autonomic arousal was measured using the Shimmer 3 GSR system, which records continuous skin conductance responses as an index of sympathetic nervous system activity. GSR data were collected throughout the experimental task to assess physiological reactivity to emotionally salient visual stimuli. Skin conductance responses (SCRs) were extracted for each stimulus condition, with higher amplitudes indicating increased autonomic arousal.

### 2.8. Laboratory Procedure

Participants attended the laboratory on two occasions (baseline and post-intervention). Each visit followed the same standardized sequence to ensure replicability. Upon arrival, participants were welcomed and screened for readiness to complete psychophysiological testing (e.g., no acute medical complaints, no factors that would prevent accurate recording such as excessive sweating or eye irritation). Participants were then seated in a quiet testing room and instructed to silence mobile devices and minimize movement during recordings. Standardized instructions were read verbatim by the research staff, including guidance on maintaining a stable posture, minimizing facial and body movements, and keeping gaze on the screen during stimulus presentation.

Next, physiological sensors were applied in a fixed order. EEG electrodes were fitted and impedance/connection quality was checked until acceptable signal quality was achieved across channels. Eye-tracking was then positioned and calibrated using a standard multi-point calibration procedure; calibration was repeated until accuracy criteria were met, and a brief validation check was performed before initiating the task. GSR sensors were placed on the palmar surface of the index and middle fingers of the non-dominant hand, and a short resting recording was obtained to confirm a stable baseline signal and the absence of recording artifacts. After all signals were verified, participants completed a brief familiarization period to reduce novelty effects and to ensure they understood the task requirements.

The experimental task began with a short baseline period (resting measurement) to capture a stable physiological reference. Participants then viewed visual stimuli presented on the monitor in a randomized order. Each trial followed the same structure: (1) a fixation marker to standardize gaze position and attentional readiness, (2) presentation of the stimulus image for a predetermined duration, and (3) an inter-stimulus interval to allow physiological responses to return toward baseline and to reduce carryover. Participants were instructed to passively view all images and to avoid deliberate emotion regulation strategies unless otherwise specified by the task instructions. The task was divided into blocks with brief, standardized breaks to reduce fatigue; sensor placement and signal quality were checked during breaks, and recalibration (eye-tracking) was performed if drift was detected.

Throughout the session, the research staff monitored signal quality in real time. Trials with major artifacts (e.g., abrupt head movement, loss of eye-tracking, or EEG saturation) were flagged for later review according to predefined criteria. At the end of the task, sensors were removed, and participants were debriefed. Any adverse events (e.g., dizziness, distress during image exposure) were documented using a standardized form, and participants were offered a short recovery period before leaving the laboratory. The same room setup, seating position, instruction script, and procedure order were used across all participants and both assessment time points to maximize procedural standardization and reproducibility.

Participants were provided with standardized information regarding the laboratory procedures, including that physiological and attentional responses to visual stimuli would be recorded; however, specific hypotheses regarding expected changes or treatment effects were not disclosed in order to minimize expectancy and demand characteristics. All instructions were delivered using a standardized script. To reduce potential confounding influences, participants were instructed to refrain from caffeine intake, nicotine use, and vigorous physical activity for at least two hours prior to testing. Sessions were scheduled at similar times of day across baseline and post-intervention assessments when possible. The laboratory environment (lighting, temperature, seating position, stimulus presentation parameters, and equipment setup) was kept constant across participants and time points. Signal quality was continuously monitored, and trials containing movement artifacts or recording instability were flagged according to predefined criteria and excluded from analysis when necessary.

### 2.9. Sample Size Calculation

An a priori power analysis was conducted using G*Power (Version 3.1.9.7; Heinrich Heine University Düsseldorf, Düsseldorf, Germany) to determine the required sample size for detecting a group × time interaction in a two-arm pre–post design (baseline vs. post-intervention). The calculation was specified as a repeated-measures framework (within–between interaction), assuming a moderate effect size (f = 0.25), a two-tailed α = 0.05, and 80% power (1 − β = 0.80) with two groups and two measurement occasions. This analysis indicated that a minimum sample close to the planned range was required; therefore, the study targeted a total enrollment of 60 participants (*n* = 30 per group). This sample size was selected to maintain adequate power while also accounting for expected attrition and potential data loss due to artifacts or unusable recordings in physiological assessments (e.g., EEG noise, eye-tracking loss, or GSR instability), consistent with the trial enrollment plan.

### 2.10. Statistical Analysis

All analyses were conducted according to a pre-specified analysis plan. Descriptive statistics were computed for all sociodemographic, anthropometric, psychometric, and laboratory variables. Distributional assumptions were evaluated using visual inspection (histograms and Q–Q plots) and normality tests; variables showing marked non-normality were analyzed using appropriate transformations or robust/non-parametric alternatives. Baseline equivalence between groups was examined using independent-samples *t* tests (or Mann–Whitney U tests, as appropriate) for continuous variables and χ^2^ tests for categorical variables.

The primary hypothesis (differential pre–post change between groups) was tested using mixed-effects models/repeated-measures ANCOVA with Time (baseline vs. post-intervention) as a within-subject factor and Group (CBT vs. control) as a between-subject factor. The key parameter of interest was the Group × Time interaction for each primary laboratory endpoint (EEG-derived indices including P300, LPP, and frontal alpha asymmetry; eye-tracking attentional bias metrics; and GSR/SCR indices), and for secondary psychometric outcomes (EDE-Q, BDI-II, BAI, YBOCS-BDD, CERQ). Where applicable, models were adjusted for clinically relevant covariates (e.g., age, BMI, baseline symptom severity) and results were reported as estimated marginal means with 95% confidence intervals. For analysis, CERQ subscales were aggregated into adaptive and maladaptive strategy composites, consistent with prior literature.

For significant effects, effect sizes were reported (e.g., partial η^2^ for ANCOVA-type models and standardized mean differences for change scores), alongside exact *p* values. To control Type I error across multiple endpoints (particularly multimodal laboratory outcomes), multiplicity was addressed using an appropriate correction strategy (e.g., false discovery rate) within outcome families (EEG, eye-tracking, GSR, and psychometrics). Missing data were handled using principled methods consistent with the model framework (e.g., mixed-model maximum likelihood), with sensitivity analyses (e.g., per-protocol analyses excluding major protocol deviations) conducted to evaluate robustness. Statistical significance was set at two-tailed α = 0.05. To ensure comparability across instruments with different scoring ranges, supplementary analyses were conducted using standardized (z-scored) outcomes; results remained materially unchanged.

## 3. Results

### 3.1. Participant Flow and Final Sample

A total of 74 individuals were initially assessed for eligibility across participating outpatient psychiatric clinics. Fifteen individuals were excluded during screening: six did not meet DSM-5 criteria for restrictive-type anorexia nervosa, three met criteria for another eating disorder (binge–purge subtype or bulimia nervosa), two had comorbid bipolar disorder, two had substance use disorders, and two were excluded due to neurological or medical conditions incompatible with laboratory-based assessments. Fifty-nine eligible participants met all inclusion criteria and were enrolled and randomized to the CBT intervention (*n* = 30) or control group (*n* = 29). Among participants included in the final analyses (*n* = 26), treatment adherence was high, with all participants completing at least 11 of the 12 scheduled sessions. Missed sessions were rescheduled whenever possible within the treatment period.

**Figure 1:**
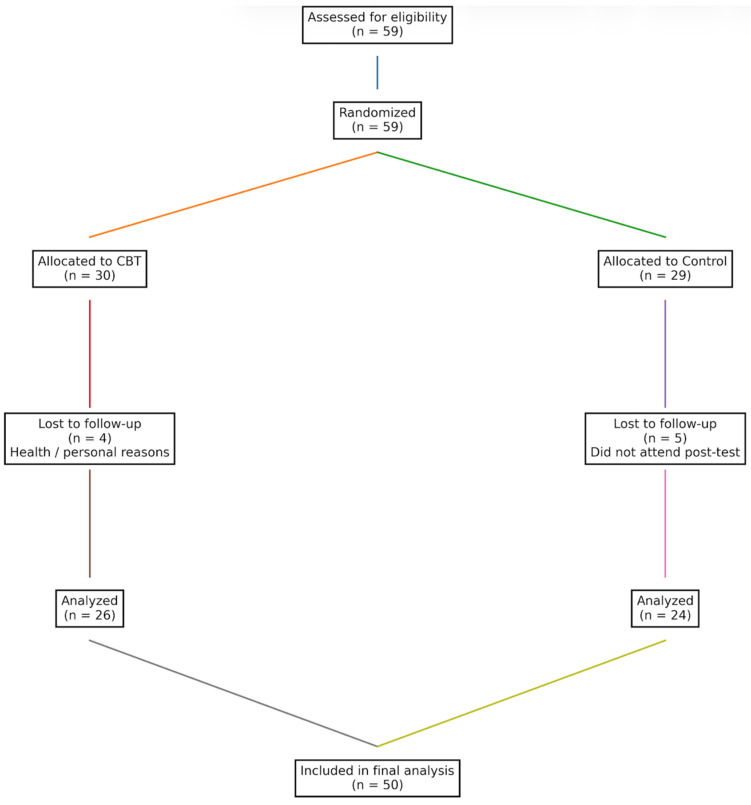
CONSORT Flow Diagram.

In Figure 1, CONSORT flow diagram illustrating participant recruitment, randomization, allocation, follow-up, and inclusion in the final analyses. A total of 59 participants were enrolled and randomized to the CBT intervention or control group. After attrition (CBT: *n* = 4; control: *n* = 5), data from 50 participants who completed both baseline and post-intervention assessments were included in the final analyses.

### 3.2. Baseline Sociodemographic and Clinical Characteristics

Baseline sociodemographic and clinical characteristics of the CBT and control groups are presented in Table 1. The two groups were comparable in terms of age, body mass index (BMI), and key sociodemographic variables. At baseline, no statistically significant differences were observed between the CBT and control groups in eating disorder symptom severity, depressive symptoms, anxiety levels, body image–related obsessive–compulsive symptoms, or cognitive emotion regulation strategies. These results indicate that the groups were well balanced prior to the intervention, supporting the validity of subsequent pre–post comparisons between groups.

All analyses were conducted on a complete-case basis, including participants who completed both baseline and post-intervention assessments (CBT: *n* = 26; control: *n* = 24).

### 3.3. Primary Psychometric Outcomes: Group × Time Effects

Group × Time effects for primary psychometric outcomes were examined using repeated-measures analyses comparing baseline and post-intervention scores between the CBT and control groups. Significant Group × Time interactions were observed for eating disorder symptom severity, depressive symptoms, anxiety symptoms, and body image–related obsessive–compulsive symptoms, indicating differential changes over time between the two groups. Specifically, participants in the CBT group demonstrated greater reductions in EDE-Q global scores, BDI-II, BAI, and YBOCS-BDD scores from baseline to post-intervention, whereas scores in the control group remained relatively stable across the same period.

For cognitive emotion regulation, a significant Group × Time interaction was also observed, characterized by an increase in adaptive cognitive emotion regulation strategies and a decrease in maladaptive strategies in the CBT group, while no meaningful changes were detected in the control group. Effect sizes for significant interactions were in the small-to-moderate range. Estimated marginal means and corresponding effect sizes for all psychometric outcomes are presented in Table 2.

Changes in anthropometric status were also examined. A significant Group × Time interaction was observed for BMI (*p* = 0.021, η_p_^2^ = 0.11). Participants in the CBT group demonstrated a modest increase in BMI from baseline (17.1 ± 0.8 kg/m^2^) to post-intervention (18.0 ± 0.9 kg/m^2^), whereas BMI in the control group remained relatively stable (baseline: 17.3 ± 0.7; post: 17.4 ± 0.8 kg/m^2^).

**Figure 2:**
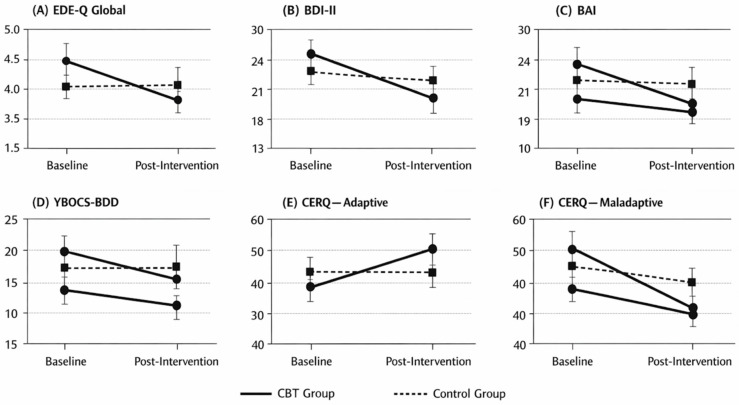
Pre-Post Changes in Primary Psychometric Outcomes by Group.

Figure 2 illustrates estimated marginal means for primary psychometric outcomes at baseline and post-intervention for the CBT and control groups. Panels depict changes in (A) eating disorder symptom severity (EDE-Q Global), (B) depressive symptoms (BDI-II), (C) anxiety symptoms (BAI), (D) body image–related obsessive–compulsive symptoms (YBOCS-BDD), (E) adaptive cognitive emotion regulation strategies, and (F) maladaptive cognitive emotion regulation strategies.

### 3.4. Neurophysiological Outcomes (EEG)

Repeated-measures analyses of EEG outcomes revealed significant Group × Time interactions for several indices of attentional and emotional processing. For P300 amplitudes, significant interaction effects were observed for both body-related and food-related stimuli, indicating a reduction in P300 amplitude from baseline to post-intervention in the CBT group, whereas changes in the control group were minimal. Similarly, LPP amplitudes elicited by body-related stimuli showed a significant Group × Time interaction, with decreased post-intervention amplitudes in the CBT group and relatively stable responses in the control group. For food-related stimuli, the Group × Time interaction for LPP amplitude did not reach statistical significance but showed a trend in the same direction.

Analyses of frontal alpha asymmetry also demonstrated a significant Group × Time interaction. Participants in the CBT group exhibited a shift toward increased relative left-frontal activity at post-intervention compared with baseline, whereas no comparable change was observed in the control group. Together, these findings indicate differential neurophysiological changes over time between the CBT and control groups, with several EEG markers showing significant effects and others demonstrating trend-level patterns consistent with the primary effects (Table 3).

**Figure 3:**
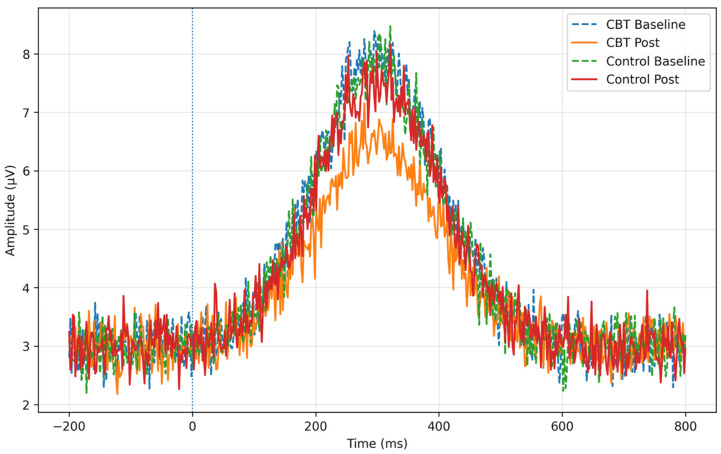
Grand-Averaged P300 ERP Responses to Body-Related Stimuli.

In Figure 3, grand-averaged EEG event-related potential (ERP) waveforms illustrating P300 responses to body-related visual stimuli at baseline and post-intervention for the CBT and control groups. A reduction in P300 amplitude following the intervention is observed in the CBT group, whereas responses in the control group remain relatively stable.

### 3.5. Eye-Tracking Outcomes

Repeated-measures analyses of eye-tracking outcomes revealed significant Group × Time interactions for multiple indices of visual attention. For body-related stimuli, significant interaction effects were observed for fixation duration, fixation count, and gaze proportion on predefined body-related areas of interest, indicating reduced visual attention to weight- and shape-salient regions from baseline to post-intervention in the CBT group. In contrast, visual attention patterns in the control group remained relatively stable across the same period.

For food-related stimuli, a significant Group × Time interaction was observed for fixation duration and gaze proportion toward high-calorie food images, with decreased attentional engagement in the CBT group following the intervention, whereas changes in the control group were minimal. The Group × Time interaction for fixation count on high-calorie food stimuli did not reach statistical significance but demonstrated a trend in the same direction, with reduced fixation frequency in the CBT group relative to controls. Overall, these findings indicate differential changes in visual attention patterns over time between the CBT and control groups, with significant and trend-level effects observed across several eye-tracking indices (Table 4).

### 3.6. Autonomic Arousal Outcomes (GSR)

Repeated-measures analyses of galvanic skin response (GSR) outcomes revealed significant Group × Time interactions for indices of autonomic arousal. For SCR amplitude, significant interaction effects were observed for both body- and food-related stimuli, indicating a reduction in physiological arousal from baseline to post-intervention in the CBT group, whereas changes in the control group were minimal. Similarly, SCR frequency in response to body-related stimuli showed a significant Group × Time interaction, with fewer responses per minute following the intervention in the CBT group compared with relatively stable response rates in the control group.

For food-related stimuli, the Group × Time interaction for SCR frequency did not reach statistical significance but demonstrated a trend toward reduced autonomic reactivity in the CBT group relative to controls. In addition, analyses of tonic skin conductance level revealed a significant Group × Time interaction, reflecting a decrease in baseline autonomic arousal at post-intervention in the CBT group, whereas tonic levels in the control group remained largely unchanged. Together, these findings indicate differential changes in autonomic arousal over time between the CBT and control groups, with significant and trend-level effects across multiple GSR indices (Table 5).

**Figure 4:**
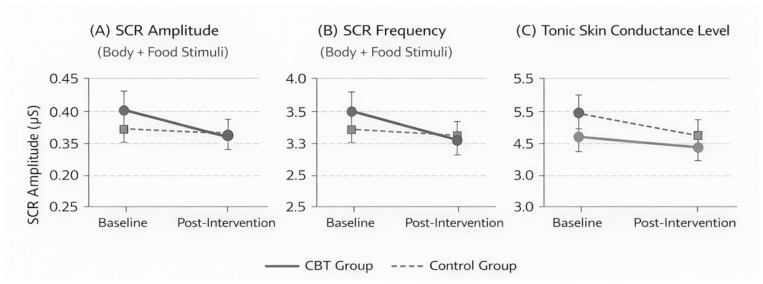
Pre-Post Changes in Skin Conductance Response (GSR) Outcome by Group.

Figure 4 illustrates pre–post changes in autonomic arousal indices derived from galvanic skin response (GSR) recordings for the CBT and control groups. Panel (A) shows changes in skin conductance response (SCR) amplitude averaged across body- and food-related stimuli, Panel (B) depicts SCR frequency averaged across stimulus categories, and Panel (C) presents tonic skin conductance level. Across all panels, participants in the CBT group demonstrated reductions in autonomic arousal from baseline to post-intervention, whereas changes in the control group were comparatively minimal. Error bars represent ±1 standard error of the mean. Statistical significance of Group × Time interactions and effect sizes for individual stimulus categories are reported in Table 5.

### 3.7. Correlation/Exploratory Analyses

Exploratory correlation analyses were conducted to examine associations between changes in psychometric outcomes and changes in neurophysiological, eye-tracking, and autonomic measures. Change scores (post-intervention minus baseline) were calculated for primary psychometric variables (EDE-Q, BDI-II, BAI, YBOCS-BDD, and CERQ indices) and for laboratory-based outcomes derived from EEG, eye-tracking, and GSR assessments.

Across the full sample, greater reductions in eating disorder symptom severity and body image–related obsessive–compulsive symptoms were modestly associated with reductions in P300 and LPP amplitudes, decreased fixation duration and gaze proportion on body-related areas of interest, and lower SCR amplitudes in response to body-related stimuli. Improvements in cognitive emotion regulation, particularly increases in adaptive strategies, showed similar patterns of association with reduced attentional and autonomic reactivity. Correlation coefficients were generally in the small-to-moderate range, and several associations did not remain statistically significant after adjustment for multiple comparisons (Table 6).

### 3.8. Adverse Events and Protocol Deviations

No serious adverse events related to the CBT intervention or laboratory procedures were reported during the study period. A small number of participants discontinued participation due to health-related or personal reasons that were unrelated to the intervention. In the control group, withdrawals were primarily due to non-attendance at post-intervention laboratory sessions and/or incomplete post-intervention psychometric assessments.

No protocol deviations occurred that required modification of the intervention, assessment procedures, or statistical analysis plan. All laboratory assessments were conducted in accordance with the predefined protocols, and data from participants who completed both baseline and post-intervention assessments were included in the final analyses.

## 4. Discussion

In this randomized controlled trial, we evaluated a 12-week cognitive-behavioral therapy intervention for women with restrictive-type anorexia nervosa, using a multimodal battery of outcome measures. The findings provided evidence of clinically meaningful improvement with CBT across both self-report measures and laboratory-based metrics, as compared to a no-treatment control group. On psychometric outcomes, participants who received CBT showed significantly greater reductions in core eating disorder symptoms, including decreased dietary restraint and body shape concerns (as reflected in EDE-Q global scores), whereas the control group’s symptoms remained essentially unchanged. The CBT group also reported improvements in general psychopathology—with moderate reductions in depressive symptoms and anxiety levels—and a marked diminishment in body image-related obsessive-compulsive behaviors (e.g., mirror checking and fixation on appearance flaws). Additionally, CBT recipients increased their use of adaptive cognitive emotion regulation strategies (such as positive reappraisal) while reducing maladaptive strategies (such as rumination), indicating enhanced coping and emotional processing post-therapy. By contrast, the control group did not exhibit any notable changes in emotion regulation. Together, these results indicate that the structured CBT program successfully alleviated eating disorder psychopathology and associated distress, outperforming the natural course of the illness over the 12-week period.

Beyond these subjective outcomes, our trial demonstrated that CBT led to significant shifts in neurophysiological, psychophysiological, and attentional processes underlying anorexia nervosa—consistent with prior theoretical models proposing that eating disorder psychopathology is maintained by interactions between affective salience networks and rigid cognitive control systems [7,8]. On EEG measures, we observed a reduction in the amplitude of the P300 event-related potential to both body-related and food-related stimuli in the CBT group (with no such reduction in controls). The P300 indexes attention allocation and salience; thus, a lower P300 after treatment suggests that disorder-relevant cues (such as high-calorie foods or body images) became less attention-grabbing or emotionally salient following therapy. Similarly, the late positive potential (LPP), a brain waveform reflecting sustained emotional attention, was significantly attenuated for body-image stimuli post-CBT, indicating a decrease in prolonged emotional arousal to seeing one’s body or shape. A trend toward reduced LPP to food stimuli was also noted. These findings align with the idea that successful therapy can dampen the hyper-responsivity to threatening stimuli often seen in AN. Similar reductions in P300 and LPP amplitudes following psychological or cognitive reappraisal interventions have been reported in ERP studies examining emotional salience processing [21,23], supporting the interpretation that modulation of these components reflects changes in affective appraisal rather than mere habituation. This is consistent with prior evidence that patients with AN initially show heightened attentional [35] and neural responses to food cues (e.g., an early hypervigilance) followed by avoidance [36]; after CBT, such extreme responses were less evident. We also found a significant change in frontal alpha asymmetry: patients moved toward relatively greater left-frontal EEG activity after CBT, a pattern generally associated with positive affect and approach-oriented emotional states. In depression and anxiety literature, increased left-frontal activation is linked to improved mood and reduced avoidance behavior [37], suggesting that our participants experienced a shift toward a more adaptive emotional regulation profile. No comparable EEG changes occurred in the control group, highlighting that these neural modifications were specifically related to the therapeutic intervention.

Convergent changes were seen in the eye-tracking data, reflecting altered visual attentional biases after CBT. Prior to treatment, individuals with AN characteristically display abnormal attention patterns, such as excessive focus on perceived “flawed” body areas and heightened vigilance toward high-calorie food cues. After the 12-week CBT program, our participants showed significantly reduced fixation duration and frequency on weight- and shape-related body regions when viewing images. In other words, they spent less time staring at “problematic” body areas (both in self-images and others’ bodies) compared to their pre-treatment behavior. This suggests a decrease in body-related attentional fixation, which could be interpreted as reduced body dissatisfaction or less preoccupation with perceived defects. The CBT group also demonstrated reduced gaze time allocated to high-calorie food images post-treatment, implying a mitigation of the threat or anxiety these images once elicited. Previous eye-tracking interventions targeting attentional bias in anorexia nervosa have demonstrated that modifying gaze allocation patterns is associated with symptom improvement [38], suggesting that attentional recalibration may represent an active therapeutic mechanism rather than a secondary consequence of symptom reduction. Notably, before therapy, such high-calorie stimuli likely captured attention due to the internal conflict they generate (attractiveness of food vs. fear of eating). After therapy, patients may experience less internal conflict or fear, allowing them to disengage more readily from food cues instead of fixating. The control group’s eye-tracking metrics remained relatively stable over the same period, reinforcing that the changes in the CBT group reflect treatment-specific effects. These outcomes are in line with literature linking attentional bias to clinical state in eating disorders [38]—for instance, greater symptom severity correlates with stronger attentional bias toward food and body stimuli [39]. Therefore, the reduction in such biases in our CBT-treated patients is a positive sign, potentially indicative of cognitive–emotional recovery. It suggests that CBT helped recalibrate the patients’ attention, steering it away from pathological preoccupations (body flaws, “forbidden” foods) and thereby breaking a maintenance mechanism of the disorder.

Changes in autonomic arousal further corroborated the therapy’s impact. Galvanic skin response (GSR) measures revealed that CBT participants had significantly lower skin conductance reactivity to both body and food cues at post-treatment, whereas control participants maintained high arousal levels. Skin conductance reflects sympathetic nervous system activation (fight-or-flight responses [40]); heightened GSR in AN is often interpreted as anxiety or stress in reaction to salient stimuli (e.g., viewing high-calorie food might provoke fear-induced arousal [41,42]). After CBT, our patients exhibited smaller amplitude skin conductance responses and fewer spontaneous fluctuations when confronted with these formerly triggering images. This attenuation of GSR implies an overall reduction in anxiety and physiological stress reactivity associated with eating and body-related stimuli [43]. Comparable reductions in skin conductance reactivity have been documented following CBT in anxiety-related disorders, where decreased autonomic responses are interpreted as evidence of successful fear extinction and cognitive restructuring [42], reinforcing the plausibility of a similar mechanism operating in anorexia nervosa. They also showed a decrease in baseline (tonic) skin conductance levels, suggesting a calmer autonomic state in general following treatment. These findings align with the notion that effective psychotherapy not only changes how patients think, but also how their bodies react. Indeed, CBT’s emphasis on exposure and cognitive restructuring may have desensitized patients to food and weight cues, resulting in less pronounced bodily fear responses. The pattern we observed—significant drops in skin conductance amplitude and frequency in the CBT group—is analogous to findings in anxiety disorder treatments, where CBT leads to diminished physiological reactivity to feared stimuli as patients learn to reinterpret or tolerate them.

Importantly, our exploratory analyses found that improvements in objective measures were correlated with improvements in clinical symptoms. Participants who showed larger reductions in eating disorder severity tended to also show greater normalization of EEG responses (e.g., bigger drops in P300/LPP amplitudes), less attentional fixation on body stimuli, and lower GSR reactivity. Likewise, increases in healthy emotion regulation strategies were associated with decreases in physiological arousal and attentional bias. Although these correlations were modest in size, they support the idea that the lab-based measures captured meaningful facets of recovery. In essence, the objective biomarkers moved in concordance with self-reported progress, reinforcing their validity as mechanistic correlates of treatment response. This correspondence between subjective and objective change is a key strength of our multimodal approach. It lends weight to the interpretation that CBT in this trial did not merely teach patients to report fewer symptoms, but truly modified the disordered cognitive-affective processes that characterize AN. As such, our study provides empirical support for the use of neurophysiological and attentional markers as indicators (or predictors) of therapeutic gains. Notably, reductions in body image-related obsessive–compulsive symptoms (YBOCS-BDD) were aligned with decreases in attentional fixation and physiological reactivity to disorder-relevant stimuli. This convergence is clinically meaningful, as obsessive preoccupations and compulsive checking behaviors are increasingly recognized as central maintaining mechanisms in restrictive-type anorexia nervosa. Heightened threat monitoring and rigid cognitive loops may manifest both psychologically (e.g., intrusive body-related thoughts, mirror checking) and physiologically (e.g., sustained salience processing indexed by P300/LPP and increased autonomic arousal). The observed parallel reductions across these domains suggest that CBT may attenuate obsessive cognitive-affective cycles not only at the subjective level but also at the level of salience processing and autonomic regulation. Given that obsessive–compulsive features in AN are often under-recognized in routine clinical practice, further research specifically examining the interaction between OCD-related symptom dimensions and neurophysiological markers may help refine personalized treatment approaches. For example, a patient whose EEG and GSR profiles do not change with therapy might be someone at risk of persisting symptoms or relapse, even if their self-report temporarily improves. This kind of insight is invaluable for advancing personalized treatment.

Our findings are generally consistent with, and extend, the existing literature on CBT for eating disorders [44]. They add quantitative evidence that CBT can be efficacious in adult anorexia nervosa—a population in which evidence-based options are limited. Previous trials of CBT (including CBT-E) for AN have reported modest success in promoting weight gain and reducing psychopathology, though often with high dropout rates and mixed results regarding superiority to other treatments [20,45,46]. In the current study, within just 12 weeks we observed moderate effect sizes (η_p_^2^ ~0.2–0.3) for improvements in eating disorder and mood measures, which is encouraging for a brief intervention in a chronic illness. This aligns with recent meta-analytic observations that while CBT’s effect on AN is not as dramatic as in bulimia, it “is probably effective in the short term”. Notably, our trial deliberately targeted not only eating symptoms but also transdiagnostic issues like mood and obsessive body image concerns; the significant improvement in these domains suggests that a broad-based CBT approach can address the wide-ranging psychopathology associated with AN. This echoes clinical accounts that CBT’s structured, multidisciplinary strategies (psychoeducation, nutritional stabilization, cognitive restructuring, exposure exercises) can produce comprehensive benefits when patients are engaged. The novelty of our work lies in demonstrating how these benefits manifest in the brain and behavior. By documenting changes in ERPs, eye gaze, and autonomic signals, we provide a more nuanced picture of recovery. For instance, while a traditional study might conclude “CBT reduced body image distress,” we can add that this reduction was accompanied by decreased limbic response and attentional engagement with body cues—evidence that therapy likely altered the emotional salience of body image internally. Our approach therefore helps bridge the gap between psychological theory and biological evidence, addressing the call for multimodal research in this field. Developmental stage is another important consideration when interpreting these findings. Although all participants met the legal criteria for adulthood (≥18 years), the age distribution skewed toward younger adults, with a substantial proportion in the 18–23 age range. Emerging adulthood is increasingly recognized as a distinct developmental period characterized by ongoing neurocognitive maturation, identity formation, and evolving autonomy. From a clinical perspective, individuals at the lower end of this age range may function developmentally more similarly to older adolescents than to fully mature adults. This is reflected in the extension of family-based treatment (FBT) models to young adults in some recent literature.

The present study employed an individual CBT-E framework; however, developmental factors may influence treatment engagement, emotion regulation capacity, and response to cognitive restructuring. It is possible that the relatively strong treatment effects observed in this sample were partly facilitated by developmental plasticity in emerging adulthood. Conversely, the absence of structured family involvement may represent a limitation for younger participants who remain embedded in family systems. Future studies should explicitly examine whether developmental stage moderates treatment response and whether integrating developmentally informed adaptations or family components enhances outcomes in younger adult populations with anorexia nervosa.

## 5. Clinical Implications

The clinical and translational implications of integrating subjective and objective outcome data are significant. From a clinical perspective, our results suggest that incorporating laboratory-based assessments into treatment evaluations could enhance the detection of meaningful change. Therapists and researchers might use tools like EEG or eye-tracking in the future to identify when a patient is truly improving versus when they might be saying the right things on questionnaires while still physiologically “locked in” to disordered patterns. For example, a persistently elevated skin conductance response to food images, despite reported fear reduction, might signal lingering anxiety that could benefit from additional intervention. Conversely, normalization in these objective measures provides reassuring confirmation of recovery processes that patients themselves might not fully articulate. In practical terms, as biofeedback and neurofeedback technologies advance, clinicians could potentially monitor certain biomarkers in real-time to personalize therapy. For example, attentional bias modification paradigms such as dot-probe–based retraining tasks, gaze-contingent eye-tracking protocols that redirect visual attention away from weight- and shape-salient regions, or virtual reality–assisted body exposure interventions may help recalibrate maladaptive salience processing. Similarly, neurofeedback approaches targeting frontal alpha asymmetry or biofeedback protocols aimed at reducing skin conductance reactivity could be integrated to directly address heightened autonomic arousal during exposure to feared stimuli. Our study demonstrates that neurophysiological and attentional metrics are sensitive to therapeutic change; thus, they could serve as early indicators of treatment response or as tools for tailoring interventions to individual profiles. Moreover, understanding the mechanisms of change can guide enhancements to CBT protocols: if we know that attentional disengagement from food cues is key, we might augment therapy with explicit attention training or mindfulness components. The integration of subjective and objective data also has research implications. It encourages a more holistic evaluation of new treatments—for instance, future trials of medication or brain stimulation for AN might incorporate similar multi-domain outcomes to fully capture benefits or unintended effects.

## 6. Limitations

Despite the strengths of this study (including its multimodal design and controlled trial methodology), several limitations must be acknowledged. First, our sample was composed entirely of women with restrictive-type AN, which limits generalizability. While AN is far more prevalent in females, about 10% of cases occur in males, who may differ in presentation and treatment response. Future research should include male patients to determine whether the CBT outcomes and mechanistic changes observed here hold true across genders. Second, participants and therapists were not blinded to treatment assignment (an inherent challenge in psychotherapy trials), raising the possibility of expectancy effects or bias in self-reported outcomes. We attempted to mitigate this by using objective measures and standardized tasks; however, knowledge of being in a therapy versus control condition could still influence how participants engage with assessments or report symptoms. Additionally, without an active placebo psychotherapy or alternative treatment group, we cannot fully disentangle the specific effects of CBT techniques from non-specific therapeutic factors (e.g., regular clinician contact or hope instillation). The control group received no intervention, which, while ethically acceptable in a short trial, means that improvements in the CBT arm might partly reflect placebo or Hawthorne effects. Third, the follow-up duration was limited to the immediate post-intervention period. We do not yet know if the gains from CBT—either the symptom improvements or the physiological changes—are maintained in the long term. Given the high relapse rates in AN, it is critical to assess whether these positive changes endure and translate into sustained recovery. Future studies should include longer-term follow-up (6–12 months or more) to evaluate the stability of both clinical remission and the persistence of normalized neurobiological markers. Another consideration is that our study’s intensive laboratory assessments, while a strength scientifically, may not be easily scalable to routine clinical practice. Specialized equipment and expertise are required for EEG, eye-tracking, and GSR, which could limit the immediate translational uptake. However, as technology becomes more portable and affordable, aspects of this multimodal assessment could be adapted for clinical monitoring. Finally, our sample size, though sufficient for detecting medium effect sizes, was modest (*n* = 50 completers). There is a need for larger trials to confirm these findings and ensure they are not idiosyncratic to our sample or setting. It would also be informative to test variants of CBT (such as adding pharmacotherapy, caregiver involvement, or novel modules) to see if the observed mechanistic changes can be accelerated or amplified. An additional consideration concerns the virtual delivery format of the CBT intervention. All sessions were conducted online via videoconferencing, which may have influenced therapeutic processes. Virtual delivery can enhance accessibility and reduce logistical barriers, potentially improving treatment adherence; however, it may also limit certain in-session behavioral interventions (e.g., direct observation of eating behaviors or in-person exposure exercises) and could affect the therapeutic alliance in ways that differ from face-to-face treatment. Although treatment adherence in the present study was high and significant improvements were observed across clinical and physiological outcomes, we cannot determine whether similar or stronger effects would have been obtained in an in-person format. Future research directly comparing virtual and face-to-face CBT-E delivery in anorexia nervosa would help clarify the impact of modality on treatment outcomes. The sample consisted of medically stable outpatients and did not include extremely underweight or inpatient-level cases, which may limit generalizability to more severe presentations. An additional limitation concerns the degree of weight restoration achieved during the intervention period. Although BMI was reassessed at post-intervention and demonstrated a statistically significant increase in the CBT group (17.1 ± 0.8 to 18.0 ± 0.9 kg/m^2^), participants remained, on average, below the normal-weight range at Time 2. Thus, while psychological and neurophysiological improvements were observed, these changes occurred in the context of partial, rather than full, weight restoration. It remains unclear whether similar neurophysiological modulation would be observed in more severely underweight or inpatient populations, or whether further normalization of BMI would produce additional shifts in salience processing and autonomic regulation. Future studies should examine the interaction between weight restoration trajectories and neurobiological outcomes over longer follow-up periods.

## 7. Conclusions

This study provides promising evidence that a 12-week CBT intervention can yield broad improvements for women with anorexia nervosa, not only alleviating self-reported symptoms but also modulating the underlying neurophysiological and attentional processes linked to the disorder. To our knowledge, it is the first RCT in AN to integrate EEG, GSR, and eye-tracking outcomes, demonstrating the feasibility and value of a multimodal lab-based approach. The CBT-treated participants experienced reductions in the psychological grip of the illness—feeling less anxious, less depressed, and less preoccupied with food and body fears—and these subjective changes were mirrored by objective signs of reduced brain hyper-reactivity, calmer physiological responses, and normalized attention patterns. These findings reinforce the role of CBT as a key therapeutic option for AN and shed light on how CBT may bring about change, supporting a mechanistic model wherein cognitive restructuring and exposure work help recalibrate both mind and body responses to previously fear-provoking stimuli. For clinicians, our results highlight the importance of addressing not just eating behaviors and weight, but also the entrenched cognitive-emotional patterns (rumination, threat sensitivity, rigid attention) that sustain AN. Interventions that successfully target those mechanisms—as CBT did here—can lead to measurable brain and behavior changes, which correspond with better clinical outcomes.

Looking ahead, we encourage future research to build on these insights. Larger trials could examine whether these neuropsychological changes serve as predictors of long-term recovery or differentiate responders from non-responders. It would also be valuable to test adjunctive interventions that explicitly leverage this knowledge—for example, incorporating attentional bias modification training or biofeedback into standard CBT, to see if that further reduces relapse. Another avenue is exploring other neurophysiological measures (such as functional MRI or hormonal stress markers) in conjunction with therapy to map a more comprehensive “systems biology” of recovery. Ultimately, embracing a multi-method approach in eating disorder research will enhance our understanding of treatment mechanisms and could pave the way for personalized medicine in AN, where both subjective experiences and objective data guide clinical decision-making. By integrating subjective and objective outcomes, clinicians and researchers can achieve a fuller picture of patient progress, ensuring that gains are not only skin-deep but reflect true healing. Our study represents an initial step toward that integrative vision, demonstrating that the marriage of psychotherapy with neuroscience tools can yield richer evidence to inform the next generation of interventions for anorexia nervosa.

## Figures and Tables

**Table 1:** Baseline Sociodemographic and Clinical Characteristics of the Study Sample.

Variable	CBT Group (*n* = 26)	Control Group (*n* = 24)	*p* Value
Age (years), mean ± SD	22.8 ± 4.1	23.2 ± 3.9	0.72
Body Mass Index (kg/m^2^), mean ± SD	17.1 ± 0.8	17.3 ± 0.7	0.41
Education level, *n* (%)			
High School graduated	6 (23.1%)	5 (20.8%)	
University continues	9 (34.6%)	8 (33.3%)	0.94
University graduated	11 (42.3%)	11 (45.9%)	
Employment status, *n* (%)			
Employed	7 (26.9%)	6 (25.0%)	
Student	15 (57.7%)	14 (58.3%)	0.98
Unemployed	4 (15.4%)	4 (16.7%)	
EDE-Q Global score, mean ± SD	4.1 ± 0.9	4.0 ± 1.0	0.78
BDI-II total score, mean ± SD	24.6 ± 8.2	23.9 ± 7.6	0.74
BAI total score, mean ± SD	22.3 ± 9.1	21.7 ± 8.8	0.82
YBOCS-BDD total score, mean ± SD	21.4 ± 6.3	20.9 ± 6.1	0.77
CERQ—Adaptive strategies, mean ± SD	43.2 ± 8.7	44.0 ± 9.1	0.73
CERQ—Maladaptive strategies, mean ± SD	51.6 ± 9.4	50.8 ± 8.9	0.76

Values are presented as mean ± standard deviation or number (percentage), as appropriate. All participants were women. *p* values refer to between-group comparisons at baseline using independent-samples *t* tests or χ^2^ tests, as appropriate. No statistically significant differences were observed between groups at baseline (*p* > 0.05 for all comparisons). To further characterize the age distribution, participants were categorized into three age bands. In the overall sample (*n* = 50), 58% were aged 18–23 years, 30% were aged 24–29 years, and 12% were aged 30–35 years. The distribution was comparable across groups: in the CBT group, 15 participants (57.7%) were aged 18–23, 8 (30.8%) were aged 24–29, and 3 (11.5%) were aged 30–35; in the control group, 14 participants (58.3%) were aged 18–23, 7 (29.2%) were aged 24–29, and 3 (12.5%) were aged 30–35. No statistically significant between-group differences were observed in age distribution (χ^2^, *p* > 0.05).

**Table 2:** Pre–Post Changes in Primary Psychometric Outcomes by Group.

Measure	CBT Group (*n* = 26) Baseline	CBT Group Post	Control Group (*n* = 24) Baseline	Control Group Post	Group × Time *p*	Effect Size (η_p_^2^)
EDE-Q Global	4.1 ± 0.9	2.6 ± 0.8	4.0 ± 1.0	3.9 ± 1.0	<0.001	0.28
BDI-II	24.6 ± 8.2	14.3 ± 7.1	23.9 ± 7.6	22.8 ± 7.9	<0.001	0.24
BAI	22.3 ± 9.1	13.1 ± 8.0	21.7 ± 8.8	20.9 ± 9.0	0.002	0.19
YBOCS-BDD	21.4 ± 6.3	13.8 ± 5.9	20.9 ± 6.1	20.2 ± 6.0	<0.001	0.26
CERQ—Adaptive	43.2 ± 8.7	51.8 ± 9.1	44.0 ± 9.1	44.6 ± 9.3	0.004	0.17
CERQ—Maladaptive	51.6 ± 9.4	42.1 ± 8.8	50.8 ± 8.9	49.9 ± 9.0	<0.001	0.22

Values are presented as mean ± standard deviation. Group × Time effects were examined using repeated-measures analyses with Time (baseline vs. post-intervention) as a within-subject factor and Group (CBT vs. control) as a between-subject factor. Effect sizes are reported as partial eta squared (η_p_^2^). CBT = cognitive–behavioral therapy; EDE-Q = Eating Disorder Examination Questionnaire; BDI-II = Beck Depression Inventory-II; BAI = Beck Anxiety Inventory; YBOCS-BDD = Yale-Brown Obsessive Compulsive Scale modified for Body Dysmorphic Disorder; CERQ = Cognitive Emotion Regulation Questionnaire.

**Table 3:** Pre–Post Changes in EEG Outcomes by Group.

EEG Outcome (Unit)	CBT Group (*n* = 26) Baseline	CBT Group Post	Control Group (*n* = 24) Baseline	Control Group Post	Group × Time *p*	Effect Size (η_p_^2^)
P300 amplitude—Body stimuli (µV)	9.84 ± 2.71	7.12 ± 2.36	9.51 ± 2.58	9.08 ± 2.64	0.003	0.17
P300 amplitude—Food stimuli (µV)	10.26 ± 2.93	8.44 ± 2.77	10.03 ± 2.74	9.81 ± 2.79	0.041	0.09
LPP amplitude—Body stimuli (µV)	7.68 ± 2.14	5.61 ± 2.03	7.41 ± 2.06	7.20 ± 2.12	0.004	0.16
LPP amplitude—Food stimuli (µV)	8.12 ± 2.33	6.74 ± 2.29	7.89 ± 2.21	7.62 ± 2.27	0.076	0.07
Frontal alpha asymmetry (FAA; log-power diff)	0.031 ± 0.083	0.094 ± 0.091	0.028 ± 0.079	0.035 ± 0.084	0.018	0.11

Values are presented as mean ± standard deviation. Group × Time effects were examined using repeated-measures analyses with Time (baseline vs. post-intervention) as a within-subject factor and Group (CBT vs. control) as a between-subject factor. Effect sizes are reported as partial eta squared (η_p_^2^). P300 = P300 amplitude; LPP = late positive potential; FAA = frontal alpha asymmetry. Reported *p* values are uncorrected; interpretation emphasizes effects surviving FDR correction within each modality.

**Table 4:** Pre–Post Changes in Eye-Tracking Outcomes by Group.

Eye-Tracking Outcome	CBT Group (*n* = 26) Baseline	CBT Group Post	Control Group (*n* = 24) Baseline	Control Group Post	Group × Time *p*	Effect Size (η_p_^2^)
Fixation duration on body AOIs (ms)	412 ± 96	305 ± 88	398 ± 91	386 ± 94	0.002	0.18
Fixation count on body AOIs (*n*)	14.8 ± 4.3	10.9 ± 3.8	14.2 ± 4.1	13.7 ± 4.0	0.009	0.14
Gaze proportion on body AOIs (%)	46.2 ± 9.7	35.4 ± 8.9	44.8 ± 9.2	43.5 ± 9.4	0.004	0.16
Fixation duration on high-calorie food (ms)	361 ± 102	289 ± 97	352 ± 99	340 ± 101	0.031	0.10
Fixation count on high-calorie food (*n*)	12.6 ± 4.9	11.1 ± 4.6	12.1 ± 4.7	11.8 ± 4.8	0.087	0.06
Gaze proportion on high-calorie food (%)	39.8 ± 10.4	33.1 ± 9.8	38.6 ± 9.9	37.9 ± 10.1	0.043	0.09

Values are presented as mean ± standard deviation. Group × Time effects were examined using repeated-measures analyses with Time (baseline vs. post-intervention) as a within-subject factor and Group (CBT vs. control) as a between-subject factor. Effect sizes are reported as partial eta squared (η_p_^2^). AOIs = areas of interest. Trend-level effects (*p* < 0.10) are reported where applicable.

**Table 5:** Pre–Post Changes in Autonomic Arousal (GSR) Outcomes by Group.

GSR Outcome	CBT Group (*n* = 26) Baseline	CBT Group Post	Control Group (*n* = 24) Baseline	Control Group Post	Group × Time *p*	Effect Size (η_p_^2^)
SCR amplitude—Body stimuli (µS)	0.42 ± 0.19	0.27 ± 0.15	0.40 ± 0.18	0.38 ± 0.17	0.006	0.15
SCR amplitude—Food stimuli (µS)	0.39 ± 0.17	0.29 ± 0.16	0.37 ± 0.16	0.35 ± 0.17	0.021	0.11
SCR frequency—Body stimuli (responses/min)	3.6 ± 1.4	2.5 ± 1.2	3.4 ± 1.3	3.3 ± 1.4	0.014	0.12
SCR frequency—Food stimuli (responses/min)	3.2 ± 1.3	2.6 ± 1.2	3.1 ± 1.2	3.0 ± 1.3	0.089	0.06
Tonic skin conductance level (µS)	5.1 ± 1.6	4.2 ± 1.4	4.9 ± 1.5	4.8 ± 1.6	0.032	0.10

Values are presented as mean ± standard deviation. Group × Time effects were examined using repeated-measures analyses with Time (baseline vs. post-intervention) as a within-subject factor and Group (CBT vs. control) as a between-subject factor. Effect sizes are reported as partial eta squared (η_p_^2^). SCR = skin conductance response; µS = microsiemens. Trend-level effects (*p* < 0.10) are reported where applicable. Reported *p* values are uncorrected; interpretation emphasizes effects surviving FDR correction within each modality.

**Table 6:** Exploratory Correlations Between Changes in Psychometric Outcomes and Multimodal Physiological Measures.

Psychometric Change Score	ΔP300 (Body)	ΔLPP (Body)	ΔFixation Duration (Body AOIs)	ΔGaze Proportion (Body AOIs)	ΔSCR Amplitude (Body)
ΔEDE-Q Global	0.34 *	0.29 *	0.38 **	0.35 **	0.31 *
ΔBDI-II	0.27	0.32 *	0.30 *	0.28 *	0.26
ΔBAI	0.22	0.25	0.27	0.24	0.33 *
ΔYBOCS-BDD	0.36 **	0.33 *	0.41 **	0.39 **	0.34 *
ΔCERQ—Adaptive	−0.31 *	−0.28 *	−0.34 *	−0.32 *	−0.29 *
ΔCERQ—Maladaptive	0.33 *	0.30 *	0.37 **	0.35 **	0.31 *

Values represent Pearson correlation coefficients (*r*). Change scores (Δ) were calculated as post-intervention minus baseline values. Positive coefficients indicate that greater reductions in symptom severity were associated with greater reductions in physiological or attentional responses. Negative coefficients indicate that increases in adaptive cognitive emotion regulation strategies were associated with reductions in physiological or attentional reactivity. Because change scores were calculated as post-intervention minus baseline values, positive correlations indicate that greater symptom improvement (more negative change) was associated with greater reductions in physiological or attentional reactivity. These analyses were exploratory. * *p* < 0.05; ** *p* < 0.01.

## Data Availability

The original contributions presented in this study are included in the article. Further inquiries can be directed to the corresponding author.
